# Hyaluronic acid–doxorubicin nanoparticles for targeted treatment of colorectal cancer

**DOI:** 10.1002/btm2.10166

**Published:** 2020-05-28

**Authors:** Daniel C. Pan, Vinu Krishnan, Alyssa K. Salinas, Jayoung Kim, Tao Sun, Sagi Ravid, Kevin Peng, Debra Wu, Md Nurunnabi, Jeffery A. Nelson, Zachary Niziolek, Junling Guo, Samir Mitragotri

**Affiliations:** ^1^ School of Engineering & Applied Sciences, Harvard University Wyss Institute of Biologically Inspired Engineering Cambridge Massachusetts USA; ^2^ Faculty of Arts and Sciences, Division of Sciences Harvard University Cambridge Massachusetts USA

**Keywords:** colitis associated cancer, colorectal cancer, doxorubicin, hyaluronic acid, polymer drug conjugates

## Abstract

Colorectal cancer, common in both men and women, occurs when tumors form in the linings of the colon. Common treatments of colorectal cancer include surgery, chemotherapy, and radiation therapy; however, many colorectal cancer treatments often damage healthy tissues and cells, inducing severe side effects. Conventional chemotherapeutic agents such as doxorubicin (Dox) can be potentially used for the treatment of colorectal cancer; however, they suffer from limited targeting and lack of selectivity. Here, we report that doxorubicin complexed to hyaluronic acid (HA) (HA‐Dox) exhibits an unusual behavior of high accumulation in the intestines for at least 24 hr when injected intravenously. Intravenous administrations of HA‐Dox effectively preserved the mucosal epithelial intestinal integrity in a chemical induced colon cancer model in mice. Moreover, treatment with HA‐Dox decreased the expression of intestinal apoptotic and inflammatory markers. The results suggest that HA‐Dox could effectively inhibit the development of colorectal cancer in a safe manner, which potentially be used a promising therapeutic option.

## 1 INTRODUCTION

Colorectal cancer is the third leading cause of cancer‐related deaths in men and in women. In 2019, over 100,000 new cases of colon cancer have been reported; it is expected to cause about 50,000 deaths. However, early diagnosis, often by screenings, resulting in the removal of colorectal pulps before they develop into cancers, and conventional therapies have resulted significant decrease in mortality.^1^, ^2^ Chemotherapies such as 5‐fluorouracil (5‐FU), triflurdine and tipiracil (Lonsurf), capecitabine (Xeloda), irinotecan (Camptosar), and oxaliplatin (Eloxatin) are frequently used in chemotherapeutic treatment of gastrointestinal (GI) cancers, including colorectal cancer.^3^, ^4^, ^5^, ^6^, ^7^, ^8^, ^9^, ^10^, ^11^, ^12^, ^13^, ^14^, ^15^, ^16^, ^17^, ^18^, ^19^ In most cases, two or more of these drugs are combined, resulting in greater efficacy.^20^, ^21^, ^22^, ^23^, ^24^, ^25^, ^26^, ^27^, ^28^ Several new chemotherapy drugs have recently been used to treat colorectal cancer, including panitumumab (Vectibix), cetuximab (Erbitux), bevaxizumab (Avastin), ramucirumab (Cyramza), and aflibercept (Zaltrap). All of these are usually administrated along with 5‐FU, irinotechan, or oxaliplatin.^29^, ^30^, ^31^, ^32^, ^33^, ^34^, ^35^, ^36^, ^37^, ^38^, ^39^, ^40^, ^41^, ^42^, ^43^, ^44^, ^45^, ^46^, ^47^, ^48^, ^49^, ^50^, ^51^ Most of these chemotherapeutic drugs, along with Regorafenib (Stivarga),^52^, ^53^ are given either intravenously or orally. Despite their efficacy, the use of these drugs is hindered by their severe side effects. For example, oxaliplatin may cause neuropathy (nerve damage) as well affecting heart rhythm in which heart muscles take longer than normal to recharge between beats. Irinotecan also induces neutropenia and diarrhea.^26^, ^54^, ^55^

Encapsulation of drugs in liposomes offers a potential means to reduce toxicity. To address this, efforts have been made to design liposomal formulations for the treatment of colorectal cancer. MM‐398 and IHL‐305, a liposomal formulation of Irinotecan, were designed to augment tumor efficacy while minimizing toxicity. Subcutaneous injection of MM‐398 showed improved efficacy while reducing toxicity in nude mice with colorectal cancer, however patients receiving one dose of MM‐398 experienced neutropenia, diarrhea, as well as vomiting and abdomen pain.^56^, ^57^, ^58^, ^59^, ^60^, ^61^, ^62^, ^63^, ^64^, ^65^, ^66^, ^67^, ^68^

Adriamycin, commonly known as doxorubicin (Dox), has been used, in combinations with other drugs, to treat many different types of cancers, such as breast, lung, ovarian, and bladder cancers, as well as neuroblastoma, leukemia, Hodgkin's and non‐Hodgkin's lymphoma. Doxorubicin encapsulated in a liposome (Doxil), administrated intravenously, has been used to treat breast cancer, ovarian cancer, Kaposi's sarcoma, and other solid tumors.^69^, ^70^, ^71^, ^72^, ^73^ However, administration of Doxil induces low blood counts and increasing risk for anemia. To our knowledge, there have been no reports of free Dox, injected intravenously, treating colorectal cancers.

Hyaluronic acid (HA), a naturally found polymer found in the body, has been shown to be useful in cancer treatment as a therapeutic. HA is able to bind to receptors on the cell membrane of tumor cells, which slows metastasis and cell migration^74^, ^75^, ^76^, ^77^ Additionally, HA can enhance the effect of conventional anticancer drugs. In a dose‐dependent manner, HA inhibits the cellular proliferation of various cancer cells including prostate, bladder, breast, melanoma, and fibrosarcoma cells.^78^ Even in the presence of escape mechanisms associated with cancer progression, the ability of HA to slow cancer growth was unaffected.^78^ HA, coupled to a conventional anticancer drug, could reduce the therapeutic dose along with reduced toxicity to healthy cells.

A combination of Dox and HA can be used as treatment. Recently, a Dox‐loaded hyaluronic acid ceramide nanoassembly releasing microspheres, administered intra‐arterially, was used to treat tumors.^79^ In this study, we assessed whether intravenous injection of Dox, complexed to hyaluronic acid (HA), can target the intestines without any affinity moieties. Our data show that HA‐complexed Dox (HA‐Dox) exhibits surprisingly high accumulation in the intestine and inhibits the progression of AOM/DSS‐induced colon cancer as well as provides a potential therapeutic effect in a safe manner.

## 2 METHODS

### 2.1 Synthesis

The drug doxorubicin (Dox), was chemically linked to 50 KDa hyaluronic acid (HA) polymer via nucleophilic acyl substitution reactions. Briefly, the polymer was dissolved in a mixture of deionized water and DMSO at a ratio of 1:1 volume. The catalyst 4‐Dimethylaminopyridine (DMAP) and the activator 1‐ethyl‐3‐3‐dimethylaminopropyl) carbodiimide hydrochloride (EDC) were added to the solvent mixture at a molar ratio of 1:1 relative to the HA monomers. Following 30 min of activation, Dox was added into the reaction mixture at molar ratios 0.4:1 relative to the HA monomer and left stirring for 24 hr. Following reaction, the product was purified by size exclusion chromatography via Sephadex G‐25 PD‐10 desalting columns (5,000 MW exclusion limit) followed by overnight dialysis (3,500 Molecular Weight Cut Off (MWCO)) against DI water. The samples were then lyophilized, stored at 4°C and reconstituted in PBS before use. The amount of Dox incorporated was measured using its respective fluorescence spectra at Ex/Em 470/590.

### 2.2 Characterization

The size and morphology of HA‐Dox were examined by transmission electron microscopy. Briefly, 2.0 μl of HA‐Dox suspensions were allowed to air‐dry on Formvar carbon‐coated cupper grids. Transmission electron microscopy (TEM) was performed on a JEOL JEM‐1400 TEM instrument, operating at a voltage of 100 kV (JEOL USA, Inc.). Particle zeta potential was measured by dynamic light scattering (DLS) on Malvern Zetasizer (Malvern). The mean particle size of HA‐Dox was estimated with a NanoSight NS3000 (Malvern Panalytical Inc., Westborough, MA).

### 2.3 In vitro hydrolysis assay

Lyophilized HA‐Dox was dissolved in PBS at 1 mg/mL DOX concentration (pH 7.4) and incubated for 5 days at 37°C in Slide‐A‐Lyzer MINI dialysis devices (10,000 MWCO). The devices were inserted in microcentrifuge tubes holding 1 ml of PBS. The 100 μl of Dox‐release medium in the microcentrifuge tubes was collected at the indicated time points and its concentration was measured via fluorescence using the TECAN plate reader. The amount of Dox released at each time point was divided with the initial amount loaded to obtain the cumulative release. All measurements were carried out in triplicate, and the results were indicated as the mean ± SD.

### 2.4 Ethics statement

All animal studies were carried out in strict accordance with the Guide for the Care and Use of Laboratory Animals as adopted by National Institute of Health, approved by Harvard University IACUC. Mice were housed in cages with free access to water and food, located in a well‐ventilated temperature‐controlled room between 18 and 23°C with relative humidity ranging from 40 to 60% under a 12‐hour light/dark period.

### 2.5 Serum and RBC collection

Healthy female balb/c mice (50–56 days) were purchased from the Charles River Laboratory (Wilmington, MA). Whole blood, collected in EDTA coated tubes (BD Microtainer) or serum collecting tubes (BD Microtainer), was spun at 1,000 g for 10 min at 4°C. Plasma and well as the buffy layer containing white blood cells and platelets, was removed; serum was stored at 4°C for 1 hr. Isolated erythrocytes (RBCs) were washed by adding ice cold 1x Dulbecco's‐phosphate‐buffered‐saline (DPBS) pH 7.4 up to 12 ml total volume and pipetting gently up and down to mix RBC extensively. RBC suspensions were centrifuged at 600*g*, 15 min, 4°C; supernatant was removed and this wash step was repeated three times.

### 2.6 Agglutination of RBC by HA‐Dox

HA‐Dox was adsorbed onto murine RBC at RT in 55% serum. Washed naïve RBC and washed RBC: HA‐Dox suspensions (1% Hematocrit) were dispensed onto a 96 U‐shaped plate and visually accessed after 24 hr at room temperature after RBC suspension had fully sedimented. Carboxylated polystyrene beads was used as a positive control.

### 2.7 Biodistrubtion

Dox, HA‐Dox_,_ HA‐AlexaFluro647, as well as HA‐Dox‐AlexaFluro647 were intravenously injected into healthy female Balb/c mice weighing between ~18 and 20 g. The biodistribution studies of Dox and HA‐Dox were performed after 0.08, 0.5, 6, and 24 hr post‐injection. At these time intervals, the blood was collected from inferior vena cava (IVC) and organs were isolated. Organs were weighed, homogenized in 10% water (w/v) and centrifuged 20,000*g* for 20 min. Homogenates (supernatant) were collected; drug was then extracted with 6x methanol, incubated for 30 min with gentle vortex, and centrifuged 20,000*g* for 20 min. Supernatant was read on a SpectraMax I3 plate reader was interpreted as a percentage of injected dose/tissue. The biodistribution studies of HA‐AlexaFluro647, as well as HA‐Dox‐AlexaFluro647 were performed after 0.08 hr and 0.5 hr post‐injection. Intestines were harvested and far red fluorescence signals were imaged using Perkin Elmer IVIS small animal imaging system.

### 2.8 Flow cytometry

HA‐Dox was adsorbed onto murine RBC at RT in 55% serum. Washed naïve RBC and washed RBC:HA‐Dox suspensions (5 μl) at 10% Hematocrit were added to 995 μl of PBS, gently vortexed, and ran on a BD LSRFortessa cell analyzer (BD Biosciences, San Jose, CA), gated at 10,000 events. Data were analyzed using FCS Express Version 6 (DeNovo Software, Pasadena, CA). Washed RBC and HA‐Dox suspensions at 10% Hemocrit were incubated at room temperature with annexin V‐647 in buffer containing 2 mM CaCl_2_ for 15 min. After incubation, an aliquot was aspirated into a BD LSRFortessa cell analyzer (BD Biosciences) for analysis, gated at 10,000 events. Results were expressed as percentage of Annexin V positive RBCs. Data were analyzed using FCS Express Version 6 (DeNovo Software). Homogenates (100 μl) from intestinal organs (duodenum, jejunum, ileum, cecum, colon), diluted in PBS (900 μl) were gently vortexed, and directly aspirated into a BD LSRFortessa cell analyzer (BD Biosciences) for analysis, gated at 10,000 events.

### 2.9 Induction of colorectal cancer

All experiments involving naive and colon cancer mice were carried out in strict accordance with Guide for the Care and Use of Laboratory Animals, adopted by National Institutes of Health, approved by Harvard University IACUC. Briefly, 50–56‐day‐old female balb/c mice were maintained in a temperature and humidity‐controlled facility under a 12 hr light/dark cycle. Water and standard diet were given ad libitum. After 1 week of acclamation, animals were subcutaneously injected once with azoxymethane (AOM) (20 mg/kg body weight). After 7 days, 2.0% dextran sulfate sodium (DSS) (Sigma‐Aldrich) was given in drinking water over 7 days followed by regular drinking water for 1 week. Saline, 105 μg Dox and 105 μg HA‐Dox was injected i.v. three times post DSS feeding period. After 1 week, mice were euthanized and intestines were harvested.

### 2.10 Histology: H&E, immunohistochemistry of cell proliferation, inflammation, and apoptosis

Immediately after mice were euthanized, intestines were harvested in and placed in 10% formalin overnight and then processed for embedding in paraffin. Paraffin‐embedded sections (5 μm) were treated with xylene; rehydrated in decreased ethanol series, and stained with hematoxylin and eosin (H and E) or immunohistochemistry and examined under a light microscope. To access the severity of AOM‐DSS‐induced colon carcinogenesis, the intensity of apoptotic and inflammatory markers was determined using IHC analysis. Colon and other intestinal tissues were immune stained with Caspase‐3 (Abcam, Cambridge MA), Bax (Novus, Littleton, CO), iNOS (Abcam), and COX‐2 (Abcam) as previously described and examined under a light microscope. Colon and other intestinal tissues were also stained with 1:200 dilution of anti‐KI67 (Abcam) and later with 1:200 dilution of anti‐rabbit secondary antibody (Abcam). The sections were then counterstained with hematoxylin and finally dehydrated and covered with coverslips.

## 3 RESULTS

### 3.1 Characterization of HA‐Dox

**FIGURE 1:**
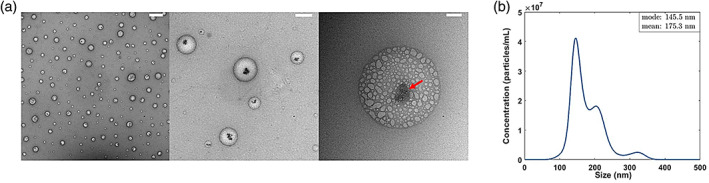
Characterization of HA‐Dox**:** (a) representative transmission electron microscopy images and (b) size distribution of HA‐Dox in PBS determined by NanoSight. Thick white line represents scale bar of 1 μm (a, left panel), 200 nm (a, middle panel), and 50 nm (a, right panel)

HA‐Dox was synthesized by conjugation of doxorubicin to HA through nucleophilic acyl substitution reaction chemistry. The morphology of HA‐Dox was examined by transmission electron microscopy (TEM). HA and Dox are spherical particles possessing a core–shell morphology (Figure 1a). Typical core size of these “nanocomplexes” was around 50 nm. Given the differences in hydrophobicities of HA and Dox, it is likely that Dox forms a hydrophobic core, surrounded by the extended shell of HA shell. TEM image, at the highest magnification, depicted a dense core (arrow) with a diffuse shell of HAs, confirming Dox incorporating within the HA‐nanocomplexes. NanoSight measurement showed that these HA‐Dox possessed a hydrodynamic size of 175 nm (Figure 1b). The broad size distribution suggests structural heterogeneity of HA‐Dox in PBS. In the presence of salt, hydrophobic interactions between Dox molecules in HA supersede ionic repulsion between the HA fibrils. This promotes self‐assembled aggregation of micelles with varying sizes, resulting in a heterogenous board distribution of HA‐Dox in PBS.

Dynamic light scattering (DLS) measurements also showed that these HA‐Dox possessed a zeta potential of −4.8 ± 1.06 mV (Figure 1b), which conveys a slightly negative charge. The negative zeta potential was likely due to the protonation of the hydroxyl groups of HA shells, which is favorable for dispersion of nanocomplexes in a biological‐relevant environment and the efficient intracellular translocation of therapeutic compounds.

### 3.2 In vitro hydrolysis

**FIGURE 2:**
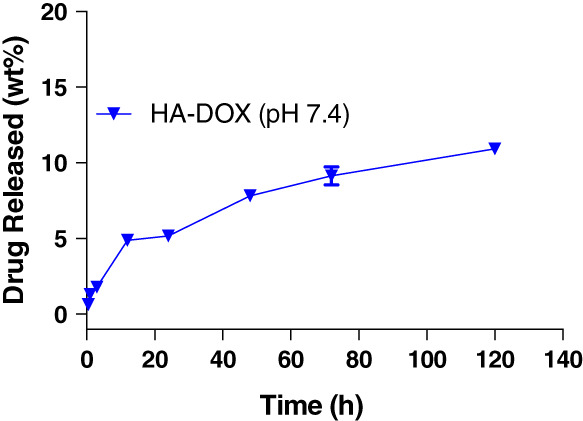
Hydrolysis of HA‐Dox**:** in vitro release of Dox from HA‐Dox in PBS at 37°C determined as wt% of initial amount of Dox conjugated to HA. Each value represents the mean ± *SD* (*n* = 3)

In vitro release rates of HA‐Dox indicated a slow and steady release of the drug from HA. Approximately, 20.6 ± 0.8 wt% Dox was released at the end of 120 hr (Figure 2). The in vitro release profile is similar to the pattern obtained previously attributing the successful incorporation of Dox and its slow release via amide hydrolysis.^80^ However, the slow release does not affect the therapeutic efficacy of HA‐Dox compared to the free Dox.

### 3.3 Pharmacokinetics of HA‐Dox

**FIGURE 3:**
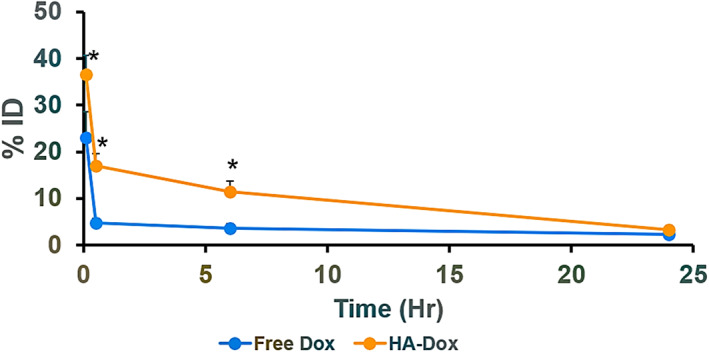
Pharmacokinetics of HA‐Dox**:** Pharmacokinetics both free Dox and HA‐Dox represented as %ID in blood at 0.08 hr, 0.5 hr, 6 hr, and 24 hr. Each data point represent means ± SEM (*n* = 3).* *p* < .05; non paired two‐tailed *t*‐test

Pharmacokinetics of HA‐Dox was evaluated in healthy Balb/c mice (105 μg of either free Dox or HA‐Dox). HA‐Dox exhibited longer circulation compared to free Dox. Dox and HA‐Dox exhibited classical elimination behavior, however, a significant difference in the elimination half‐life was found. While only 20% ID of free Dox was detected in blood 0.08 hr after injection, 40% ID of HA‐Dox could be detected in the blood at the time point (Figure 3). The difference between blood concentrations of free Dox and HA‐Dox persisted across all time points studied after 24 hr. The amount of HA‐Dox and free Dox circulating in the blood decreased 24 hr after administration (3% ID and 2% ID, respectively).

**FIGURE 4:**
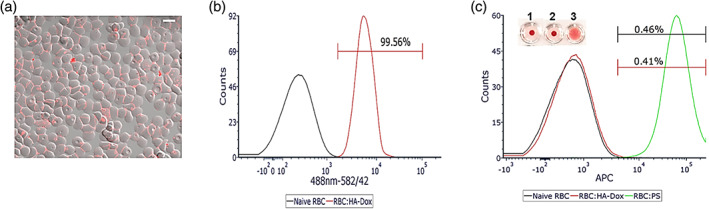
Binding and Biocompatibility of HA‐Dox on RBC**: (**a) representative confocal image, scale bar = 5 μm, and (b) flow cytometry histogram displaying percentage of RBC with HA‐Dox bound to their surface in the presence of 55% serum. (c) Representative plot displaying percentages of phosphatidylserine exposing RBC of HA‐Dox attached onto RBC. Polystyrene beads were used as a positive control. Values are means (*n* ≥ 10) ± SEM, Inset Representative agglutination image, visualized using a U‐shaped bottom plate. (1) Naïve RBC; (2) RBC:HA‐Dox; (3) RBC:Polystyrene beads

The mechanisms of enhanced persistence of HA‐Dox in blood were studied. Hyaluronic acid by itself has a blood half‐life of 3–5 min in the blood. Hence, simple association of dox with HA is not expected to render long circulation. We assessed whether association of dox‐HA with red blood cells (RBCs) may be responsible for extended circulation. Prior literature has shown that RBC‐association enhances the circulation of polymeric nanoparticles.^81^, ^82^, ^83^ HA‐Dox bound to murine RBC with high efficiency; nearly 100% of RBC exhibited attachment HA‐Dox on the surface in the presence of 55% serum (Figure 4a,b).

Adsorption of HA‐Dox onto RBCs did not induce agglutination (Figure 4c, inset), implying that HA‐Dox may not be detrimental to RBC. Furthermore, we investigated whether HA‐Dox induces exposure to phosphatidylserine, a signal released from senesced and damaged RBC that facilitates their clearance from the blood circulation. The adsorption of HA‐Dox did not induce an increase in RBCs expressing phosphatidylserine (0.41% ± 0.2) compared to naïve (0.46% ± 0.3) (Figure 4c).

### 3.4 Biodistribution of HA‐Dox

**FIGURE 5:**
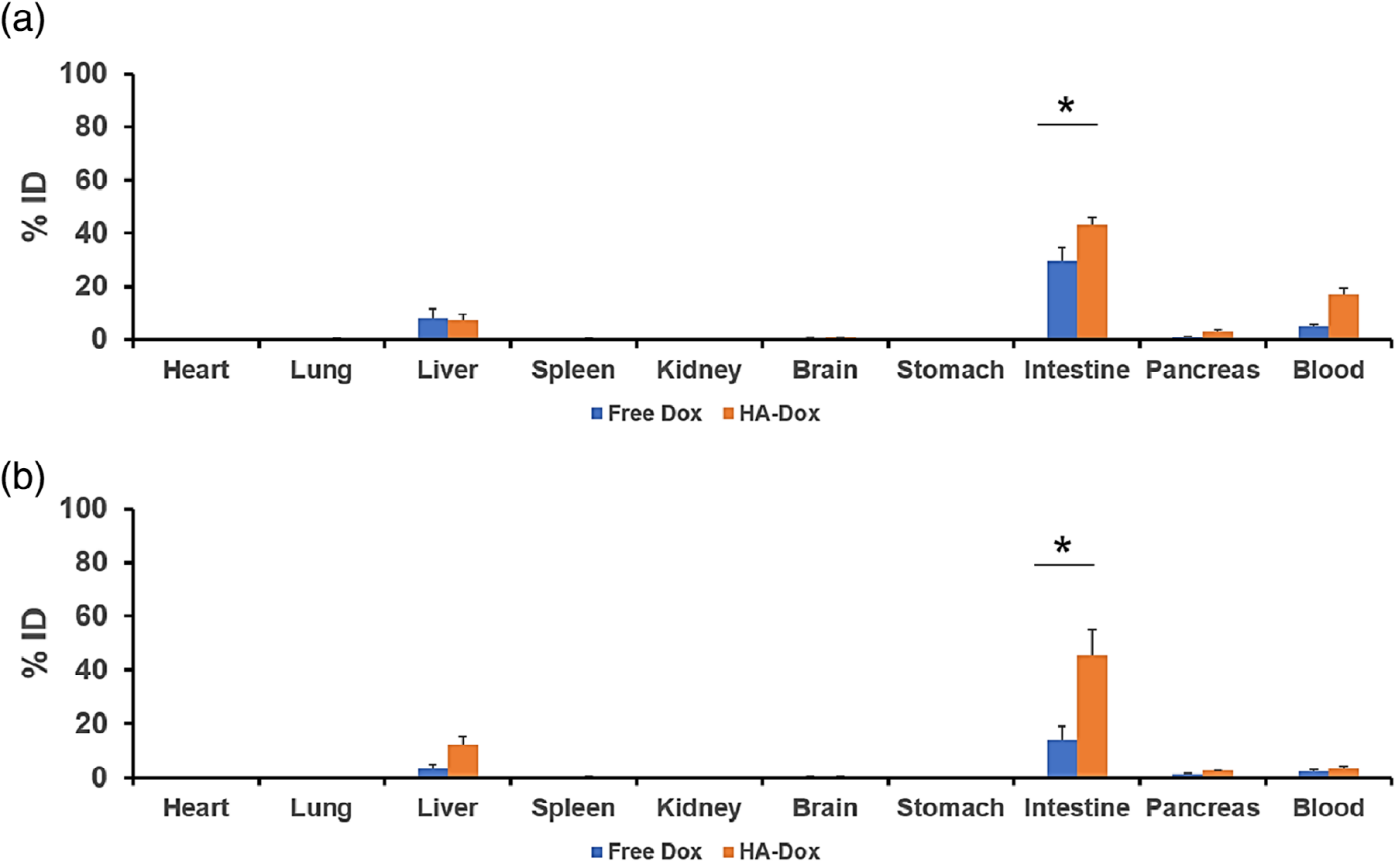
Biodistribution of HA‐Dox: Biodistribution of Dox and HA‐Dox. Bar graph represents percentages of injected dose (%ID) for free Dox and HA‐Dox following IV injection in mice at (a) 0.5 hr and (b) 24 hr. Each data point represent means ± SEM (*n* = 3).*, *p* < .05; non paired, two‐tailed t‐test

Biodistribution of Dox and HA‐Dox in mice was assessed. Significant accumulation of HA‐Dox was found in intestine (~45% ID), while only ~7% ID was found in the liver 0.5 hr after administration (Figure 5a). Minimal amounts were detected in the heart, lung, spleen, kidney, brain, stomach, pancreas, and the spleen. Free Dox accumulated in the liver (8% ID) 0.5 hr after administration and ~30% ID of free Dox accumulated in the intestine. ~5% ID of free Dox remained in circulation. There was a significant increase in HA‐Dox accumulation in the intestine compared to its free Dox counterpart (~45% ID vs ~30% ID respectively). The amount of HA‐Dox in the intestine persisted at ~45% ID for 24 hr. Similar trend was found for free Dox except that the magnitude of accumulation was at 15% ID. (Figure 5b). There was nearly a threefold increase in the intestine:blood ratio of HA‐Dox compared to free Dox.

**FIGURE 6:**
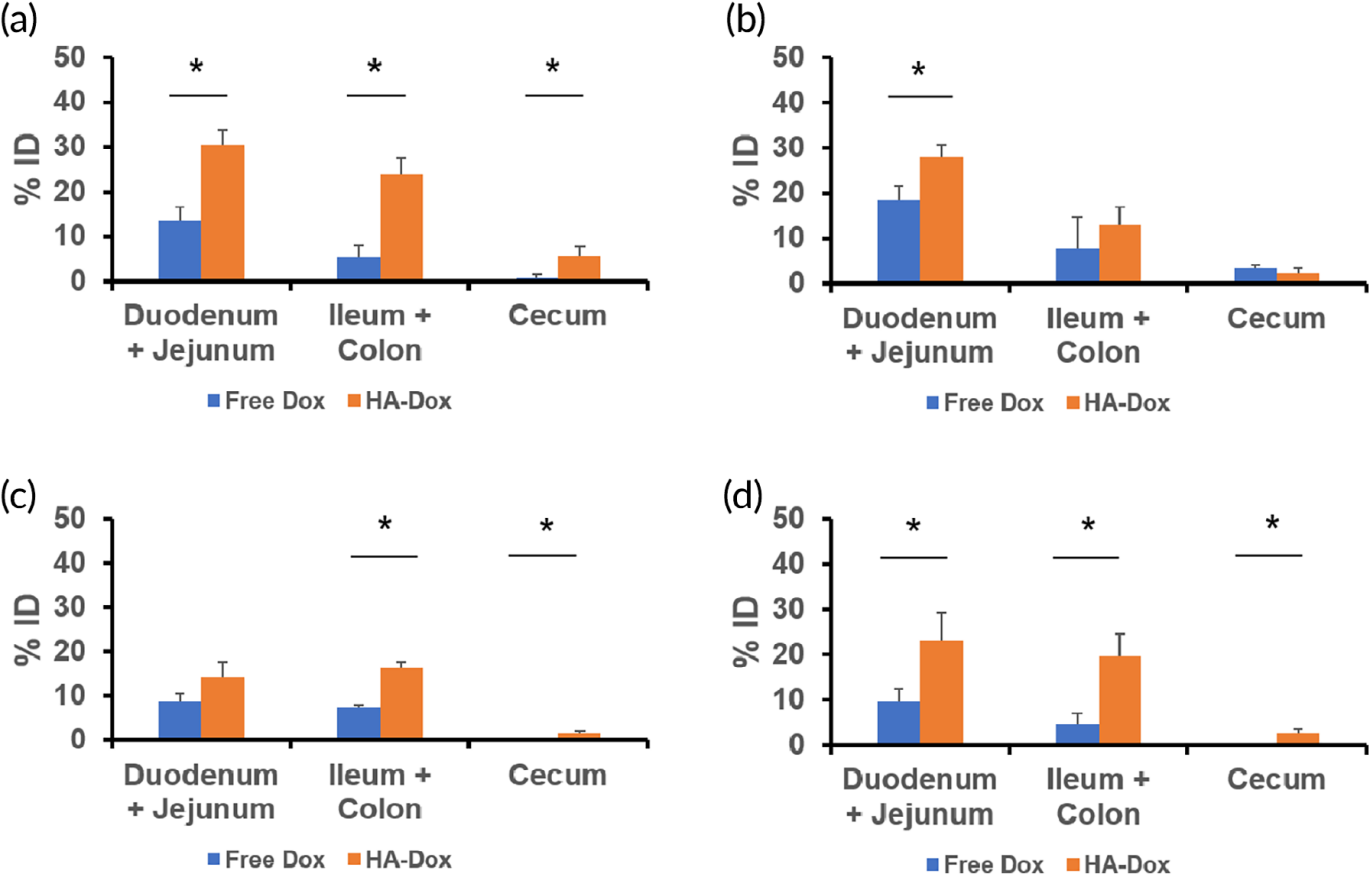
Accumulation of HA‐Dox in Intestines**:** Kinetics of HA‐Dox represented as percentages of injected dose (% ID) for free Dox and HA‐Dox following I.V. injection in mice at (a) 0.083 hr (b) 0.5 hr (c) 6 hr and (d) 24 hr in different part of murine intestines. The amount of HA‐Dox at 24 hr Each data point represent means ± SEM (*n* = 3). * *p* < .05; non paired, two‐tailed *t*‐test

Within the intestines, most HA‐Dox as well as free Dox was found to accumulate in the duodenum and jejunum after 0.08 hr. Unlike free Dox, a significant accumulation of HA‐Dox was also observed in the ileum, colon, and cecum (Figure 6a). Compared to 0.08 hr, the amount of HA‐Dox in the duodenum and jejunum remained fairly constant after 0.5 hr (Figure 6b), 6 hr (Figure 6c), and 24 hr (Figure 6d). This trend was also observed in the ileum and colon. The accumulation of HA‐Dox in the cecum decreased over time. After perfusion at 24 hr, the amount of HA‐Dox significantly decreased in all parts of the intestines (Figure S1), suggesting that the majority of HA‐Dox accumulates inside the blood vessels of the intestine.

To our knowledge, there have been no reports of free Dox or any Dox complexes accumulating in the intestines via intravenous injection. To confirm HA‐Dox accumulates in the intestines, duodenum and jejunum, ileum and colon, and cecum homogenate were obtained from mice 24 hr after IV administration and analyzed using a BD LSRFortessa cell analyzer. For each intestinal homogenate, a mean fluorescence intensity (MFI) shift in the Dox channel (488 nm‐582/42) compared to the noninjected mice was observed (Figure S2). Furthermore, HA‐Dox accumulation (displayed as bright green dots) in each intestinal tissue, 0.05 hr and 6 hr after intravenous administration, was also confirmed using confocal microscopy (Figure S3).

To assess the role of HA in targeting of HA‐Dox in the intestine, localization of AlexaFluro647‐labeled HA was measured. Signal intensities were observed in the stomach 0.08 hr after administration. Some signal was observed in all parts of the intestine (duodenum, jejunum, ileum, and the colon), suggesting that HA accumulates in the intestines (Figure S4a). Although at a reduced level, signal could still be observed in the duodenum, colon, and different parts of the jejunum and ileum 0.5 hr postinjection (Figure S4b). Similar to HA‐AlexaFluro647, strong signal was observed in the stomach for HA‐Dox‐AlexaFluro647 and low signal were observed in the cecum and colon at both time points. No signal was detected in the duodenum, jejunum, and ileum (Figure S5a). At 0.5 h post injection, however, stronger signal was detected in the duodenum and the beginning of the jejunum and no signal was detected in the ileum, cecum, or colon (Figure S5b). The presence of Dox was verified using a BD LSRFortessa cell analyzer. For each duodenum and jejunum intestinal homogenate, there was a mean fluorescence intensity (MFI) shift in the Dox channel (488 nm‐582/42) compared to non‐injected mice (Figure S5c). Ileum, colon, and cecum homogenates displayed a small shift (Figure S5d,e) in the Dox channel. These results suggest that both HA and Dox are responsible for the intestinal accumulation of HA‐Dox.

### 3.5 Effect of Dox and HA‐Dox on early signs of chemical induced colon cancer

**FIGURE 7:**
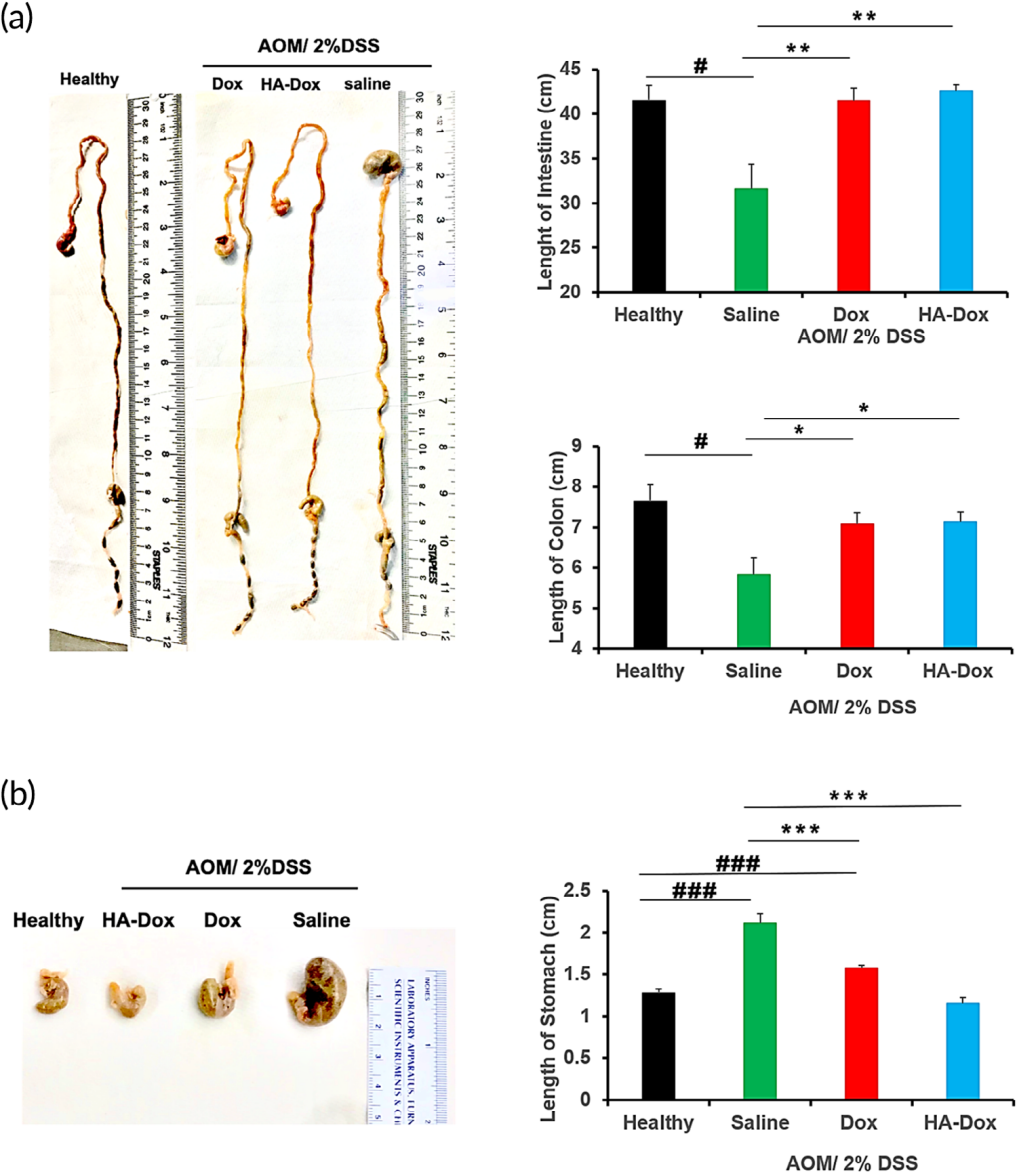
Preventive effect of HA‐Dox on inflammation‐linked carcinogenesis in AOM/DSS mouse models: (a) representative intestines and (b) stomachs are shown. Healthy mice were not injected with AOM and received distilled water only. Healthy; *n* = 4 mice; saline, Dox, and HA‐Dox, *n* = 6–7 mice; Error bars, SEM. ###, *p* < .001 versus healthy; ***p < .001 versus saline. Each data point represent means ± SEM (*n* = 3).*, *p* < .05; non paired, two‐tailed *t‐*test

The therapeutic potential of HA‐Dox was tested using AOM‐DDS‐induced colon cancer. Exposure to AOM‐DDS induced colon cancer‐like symptoms including significant shortening of the colon and intestines. Treatment of mice with saline alone did not revert the length reduction (Figure 7). In contrast, Dox‐treated or HA‐Dox treated mice exhibited lengths comparable to healthy mice. Saline treated AOM‐DDS mice also exhibited significantly larger stomachs (Figure 7), compared to their free Dox and HA‐Dox counterparts. This swelling is most likely due ascites, a pathological fluid accumulation within the peritoneal cavity that associated with colon cancer.

**FIGURE 8:**
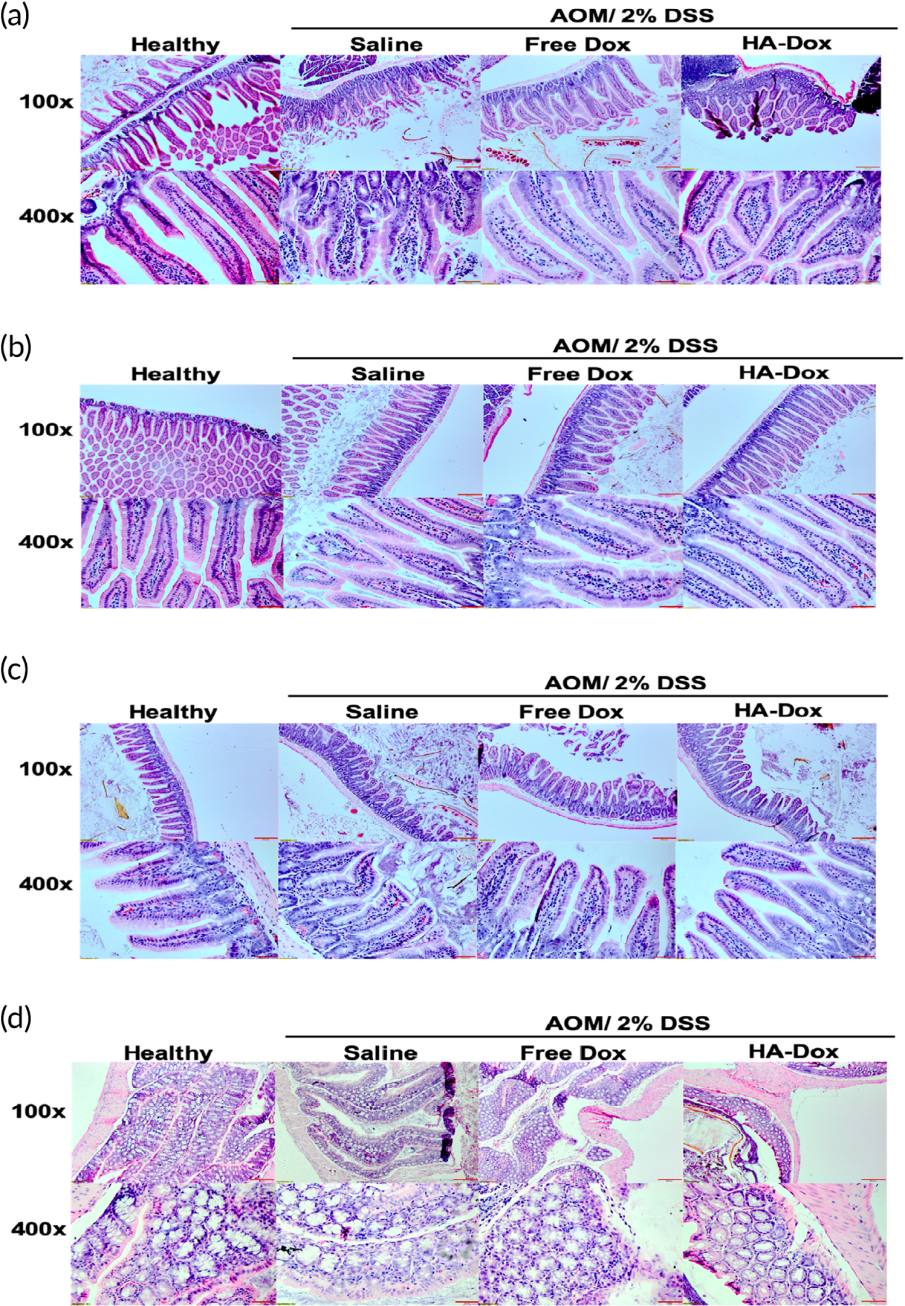
Histological analysis of murine intestinal tissues induced with chemical induced colon cancer: Histology of intestinal tissues (a) duodenum, (b) jejunum; (c) ileum, and (d) colon treated with azoxymethane and dextran sulfate treated with saline, Free Dox, and HA‐Dox, determined by H and E staining; Representative images were taken at ×100 (top) and ×400 (bottom)

To investigate the effect of HA‐Dox in chemical‐induced colon cancer, we first performed histological analysis of AOM/DSS mice intestinal tissues. Microscopic evaluation of H&E stained small intestine and colon sections of AOM/DSS‐treated mice displayed increased intestinal injuries compared to healthy mice (Figure 8). Saline injected AOM/DSS mice exhibited substantial damaged intestinal villi and villi atrophy, which is exhibited by the erosion of the villi, resulting in a flat surface. The degree of villi atrophy is graduated. AOM/DSS mice injected with free Dox displayed a milder form of villi atrophy, while the small intestine of the HA‐Dox‐treated AOM/DSS mice were covered with long villi that were almost the same length as the villi observed in healthy mice. Furthermore, villi of AOM/DSS mice also displayed large tears, compared to the healthy counterparts. Similar to villi atrophy, the length of these tears is graduated. The villi in saline injected AOM/DSS mice displayed large tears while villi in mice treated with free Dox displayed smaller tears. HA‐Dox‐treated AOM/DSS mice had villi that had very small tears and appear to have the lining of the villi almost as intact as that of healthy mice.

### 3.6 The effect of Dox and HA‐Dox on intestinal excessive cell proliferation of mice with chemical induced colon cancer

**FIGURE 9:**
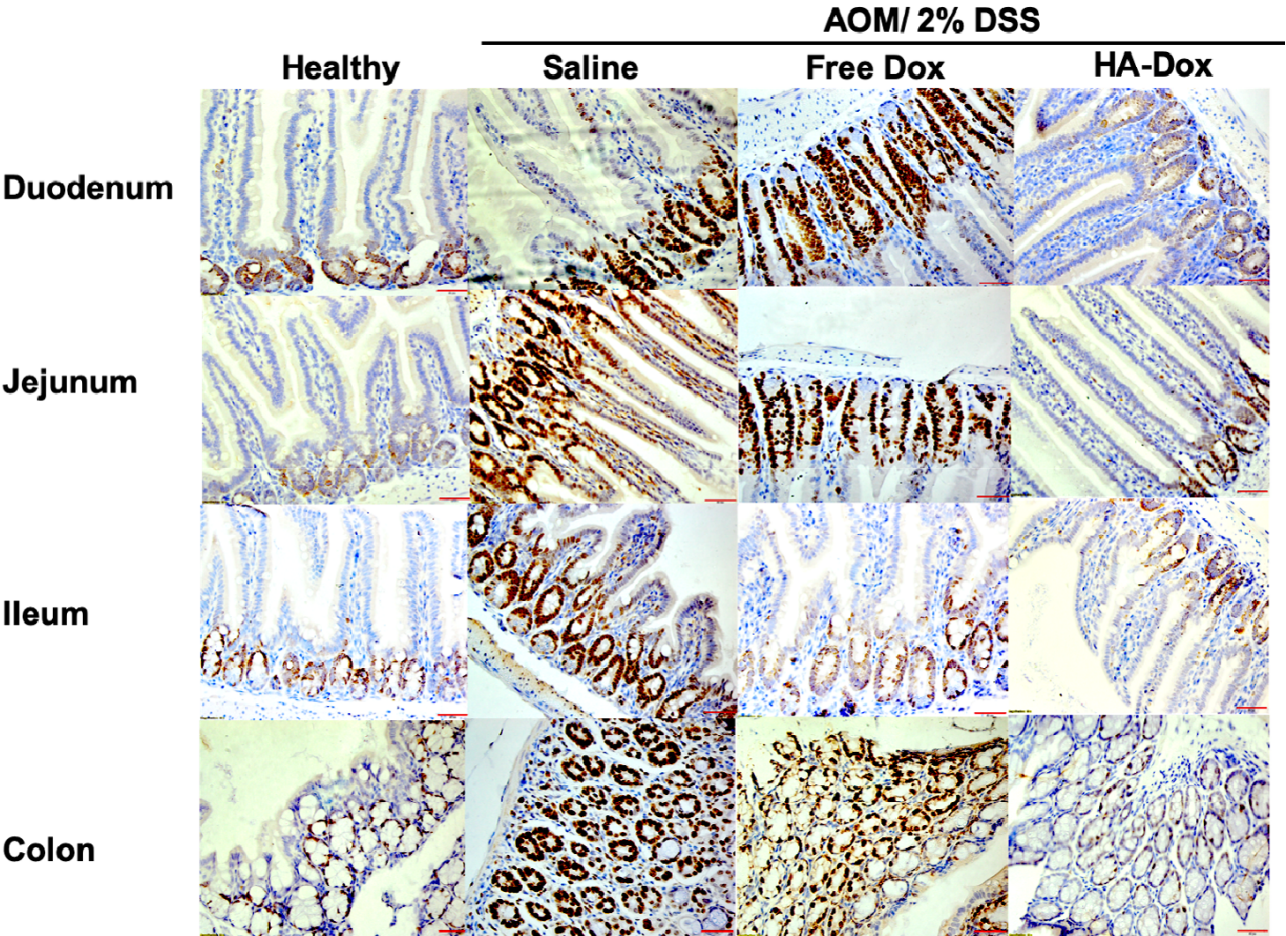
Cell proliferation analysis of murine intestinal tissues induced with chemical induced colon cancer: Immunohistochemistry of intestinal tissues treated with azoxymethane and dextran sulfate treated with saline, Free Dox, and HA‐Dox, determined by Ki67. Representative images were taken at ×400. Red scale bar: 50 um

To evaluate the effect of HA‐Dox on the proliferation and differentiation ability of intestinal crypt cells, Ki67 were assessed by immunohistochemistry staining (Figure 9). The expression of Ki67 is strongly associated with tumor cell proliferation and growth. Many studies have suggested that the overexpression of Ki67 or the loss of proliferation control appear to be linked to colon cancer. Overall, the number of Ki67 positive cells (stained brown) in duodenum, jejunum, ileum, and colon of AOM/DSS mice injected with HA‐Dox were markedly lower compared to the intestinal tissues in AOM/DSS mice injected with either saline and Dox, although the Ki67 expression levels in AOM/DSS mice injected with Dox was lower than AOM/DSS injected with saline. The number of Ki67 positive cells in intestinal tissues of AOM/DSS mice injected with HA‐Dox was very comparable to levels of Ki67 positive cells in the intestinal tissues of healthy mice. It is important to note that there is a baseline level of cell proliferation associated with the constantly regenerating epithelial layer of the intestinal villi. These results indicate that HA‐Dox might be helpful in maintaining differentiation and proliferation ability of intestinal crypt cells in colon cancer.

### 3.7 The effect of Dox and HA‐Dox on intestinal inflammation in mice with chemical‐induced colon cancer

The effect of HA‐Dox on the inflammation in AOM/DSS mice was also assessed in terms of (cyclooxygenase‐2) COX‐2 and inducible nitric oxide synthase (iNOS) expression levels by immunohistochemistry (Figure S6). Although there were COX‐2 positive cells in healthy/normal intestinal tissues, there was a dramatic increase in COX‐2 positive cells, stained brown, in AOM/DSS mice intestinal tissues compared to healthy intestinal tissues. Intestinal tissues of the healthy group had normal architecture, although there were cyclooxygenase‐2 positive cells exhibited in the mucosa, particularly prevalent in the epithelial lining encapsulating the villi. AOM/DSS mice injected with HA‐Dox had a lower expression of COX‐2 in duodenum, jejunum, ileum, and colon compared to the AOM/DSS mice injected with saline and free Dox. A higher rate of cell proliferation tended to have an increased expression of COX‐2. These findings are significant in HA‐Dox and may play a role in the management of COX‐2 expression, suggesting it might have COX‐2 inhibitor properties. Similarly, iNOS positive cells were also predominantly prevalent in the epithelial lining surrounding the villi. Interestingly, there was no significant difference in the expression of iNOS in all intestinal tissues in AOM/DSS mice injected with saline, free Dox, and HA‐Dox. All three of these groups were comparable to iNOS expression levels in the intestines present in the healthy mice.

### 3.8 The effect of Dox and HA‐Dox on intestinal apoptosis in mice with chemical‐induced colon cancer

There have been studies that have shown that Dox induces apoptosis by activation of caspase 3 in cultured cardiomyocytes in vitro and rat cardiac ventricles in vivo. It is unclear whether administration of Dox can cause the increased activation of caspase‐3 in the intestines with the concomitant apoptosis and shedding of villus in healthy or sick mice. To specifically address the type of cell death responsible for intestinal epithelial cell (IEC) shedding and loss from the villus, we performed IHC for active caspase‐3 as well as Bax2 in healthy and AOM/DSS mice (Figure S7). Interestingly, caspase‐3‐activation in the villi of the duodenum, jejunum, and ileum was strongly reduced after injection with either Dox or HA‐Dox compared to saline in AOM/DSS mice so that the level of caspase‐3‐activation in treated mice was comparable to levels in healthy mice. In contrast, there was no significant difference in the levels of caspase‐3‐activation in the colon between saline, Dox, and HA‐Dox treated AOM/DSS mice though caspase‐3‐activation was lower in the colon of healthy mice. Interestingly, there was no significant difference in Bax‐2 levels between saline, Dox, and HA‐Dox treated AOM/DSS mice and healthy mice, although Bax‐2 levels were elevated in the duodenum of HA‐Dox treated AOM/DSS.

## 4 DISCUSSION

Doxorubicin has been used to treat cancers such as leukemia, lymphoma, as well as breast cancer, lung cancer, and thyroid cancer. However, there are no reports of the use of doxorubicin to intestinal and colon cancers. To our knowledge, this is the first report that intravenous injections of doxorubicin variants can target the intestines. There are many studies have shown that drugs taken orally are absorbed systemically from the small intestine, making oral drug delivery to be the traditional choice for intestinal targeting. However, oral administration of doxorubicin remains a challenge for several reasons. Doxorubicin undergoes acid hydrolysis in the stomach and any remaining doxorubicin reaching the intestine is discharged by P‐glycoprotein, found in the epithelial cells lining the small intestine and colon. Thus, doxorubicin exhibits low oral bioavailability and traditional drug delivery pathway to the intestines is hindered. It is only available on the market as intravenous formulations, often laden with cardiotoxicity and a drastically reduced ability to specifically target the intestines and colon. However, our variant of doxorubicin conjugated to hyaluronic acid is biocompatible and does not exhibit the issues present in the current intravenous injection therapy.

The mechanism that enables HA‐Dox to target and remain in the intestine after being intravenously injected is unclear; however, the presence of CD44 and RHAMM in the intestine may offer insight into the phenomenon. HA‐Dox, a 150 nm nanoparticle‐like complex, was observed 0.08 hr (5 min) after administration in the blood, however, over time, HA‐Dox was cleared from the bloodstream. HA‐Dox is able to absorb onto RBC in the presence of serum; however, it detaches quickly. Unlike most nanoparticles that are able to adsorb onto RBC,^83^, ^84^ HA‐Dox does not accumulate in the lungs, rather it accumulates in the intestines. Interestingly, a large amount of HA‐Dox was found in the small intestine at 0.08 hr postinjection, in particular, the duodenum and the jejunum. Furthermore, approximately, the same amount of HA‐Dox was still present after 24 hr postinjection. Previous studies have shown that CD44 (a membrane glycoprotein) and receptor for hyalurone‐mediated motility (RHAMM) two receptors in which hyaluronic acid (HA) can bind to are found in the intestines.^77^, ^85^, ^86^, ^87^, ^88^, ^89^, ^90^, ^91^, ^92^, ^93^, ^94^ CD44 is normally expressed in lower crypt epithelium of the intestinal mucosa, localized to the basolateral membrane of the cells. This may play a role in the generation and turnover of epithelial cells. In addition, CD44 was found in the intestinal lamina propria, in particular on stromal cells, macrophages, and lymphocytes. Comparable to the results found in mice, CD44 is overexpressed in human colorectal cancer.^77^, ^85^, ^86^, ^87^, ^88^ Akin to CD44, there have been studies that have shown that RHAMM protein expression is found in organs with high cell turnover, such as the small intestine and colon, in particular the base of the intestinal crypts.^77^, ^89^, ^90^, ^91^, ^92^, ^93^, ^94^ Taken together, the presence of CD44 and RHAMM offers a potential supposition as to why the HA‐Dox targets and remains in the small intestine.

In addition, there have been studies focusing on the inter‐ and intra‐cellular interactions between hepatic cells and nanoparticles as well as the elimination of nanoparticles through the hepatobiliary system.^95^ In our study, HA‐Dox was found in the liver. Since no HA‐Dox was found in the stomach at any time points in our biodistribution study, it is plausible that HA‐Dox traveled to the portal vein in the liver after administration and transcytosed through the hepatocytes and entered the bile duct into the duodenum of the small intestine, where we saw large accumulation. The AlexaFluro647‐labeled HA and HA‐Dox found in the stomach could be potentially mediated by the fluorophore itself or interference from the food in the measurements.

We thus analyzed whether intravenous injection of HA‐Dox in AOM/DSS mice could be used to prevent colorectal cancer progression. The accumulation and therapeutic effects of HA‐Dox in the intestinal tissues are probably enhanced by both an increase in blood flow and in vascular permeability; both being well documented in inflammatory bowel disease (IBD). Dox slows or stops the growth of cancer by blocking topoisomerase 2. There have been studies that have shown HA a promising anti‐cancer agent. There have been studies that have shown that HA can act as inhibitor of tumor vascularization, tumor metastasis, cancer formation, and cancer growth. Mechanism of its action include either to promote the creation of a capsule from the connective tissue, resulting in the encompassing of the tumor, proteolytical activation of CD44, or binding to the cell membrane of the tumor cells.^96^ Intriguingly, after multiple injections of HA‐Dox in AOM/DSS mice, the length of the small intestine and colon as well as the size of the stomach were similar to those of the healthy mice. Our data revealed that cell proliferation in the small intestine and colon crypts, inflammation, and apoptosis in the villi were reduced in the HA‐Dox AOM/DSS‐treated mice, compared to the saline treated AOM/DSS mice, suggesting the importance of HA‐Dox as a prophylactic therapeutic capable of curbing the spread of cancer in the intestine.

There is evidence that a link between inflammation and colorectal cancer exists.^97^, ^98^, ^99^, ^100^, ^101^, ^102^, ^103^, ^104^, ^105^, ^106^, ^107^ Many studies have shown that pro‐inflammatory mediators such as cyclooxygenase‐2 (COX‐2) and lipoxygenase pathways may lead to tumor cell proliferation, growth, thus promoting colorectal cancers. Anti‐inflammatory agents such as COX2 inhibitors, as well as iNOS inhibitors, suppress colorectal cancer by inhibiting inflammatory pathways.^108^, ^109^, ^110^, ^111^, ^112^, ^113^, ^114^, ^115^, ^116^, ^117^, ^118^, ^119^, ^120^, ^121^, ^122^, ^123^, ^124^ COX‐2 inhibitors (e.g., rofexoxib, celecoxib, and valdecoxib), subclass of nonsteroidal anti‐inflammatory drugs, reduce the production of prostaglandins, chemical that promotes inflammation.^114^, ^125^, ^126^ By providing anti‐inflammatory benefits, it allows the COX‐1 enzyme to retain its gastroprotectivity functions. Our findings suggest that HA‐Dox functions similarly to COX‐2 inhibitors in this regard.

There are many reports that show that chemotherapy causes extensive damage to the DNA present in the intestinal cell wall. Researchers have been looking into reducing the damage done to the intestinal cell walls from chemotherapy, as it would render the treatment more bearable and allow for a higher rate of implementation of chemotherapy.^127^, ^128^, ^129^, ^130^, ^131^, ^132^, ^133^, ^134^, ^135^, ^136^ Considering this, in our study, it is unclear whether HA‐Dox induces apoptosis in the intestine due to the continuous shedding of the intestinal epithelium. As this is the most rapidly renewing tissue in the body, undergoing almost complete cellular turnover in as little as a few days, it is difficult to distinguish between normal cell death and apoptosis that is symptomatic of intestinal cancer. In IBD, excessive cell death and apoptosis is observed in the colon and ileum epithelium. The development of IBD‐related colorectal cancer (CRC), chronic inflammation is a major risk factor for gastrointestinal malignancies development in IBD patients. There has been evidence over the decade that show a link between chronic intestinal inflammation and CRC thorough a series of events; from the development of early dysplasia to low grade dysplasia to high‐grade dysplasia to eventually converting to invasive adenocarcinoma.^137^, ^138^, ^139^, ^140^ The development of extra‐intestinal malignancies has also been shown in IBD patients. Currently, therapies to treat IBD diminish the mucosal inflammatory response.^138^ Our findings suggest HA‐Dox lowers inflammation levels in all parts of the intestine, stopping the further development of CRC. Future studies should focus on assessing toxicity of HA‐Dox in detail including cardiac toxicity. Future studies should also include a detailed evaluation of the mechanisms of anti‐inflammatory properties of HA‐Dox.

In conclusion, our study revealed that HA‐Dox may be an effective therapeutic agent to prevent or reduce the risk of colorectal cancer development, particularly for people who already suffer from IBD.

## Supporting information


**Supplemental Figure 1 Accumulation of HA‐Dox in Perfused Intestines:**. The amount of HA‐Dox represented as percentage of injected dose (%ID) for free Dox and HA‐Dox following I.V. injection in mice at 24 h after perfusion compared to no perfusion remaining in different parts of murine intestine. Each data point represent means ± SEM (n = 3).*, *p* < 0.05; non paired, two‐tailed *t*‐test.
**Supplemental Figure 2: Accumulation of HA‐Dox in Intestines:** Representative flow cytometry histograms portraying the presence of HA‐Dox following I.V. injection in mice at 24 h in (A) Duodenum and Jejunum (B) Ileum and Colon and (C) Cecum homogenates. Dox spiked in was used as a positive control.
**Supplemental Figure 3: Accumulation of HA‐Dox in Intestines:** Representative confocal images portraying the presence of HA‐Dox represented by dots following I.V. injection in mice at (A) 0.08 h and (B) 6 h in Duodenum and Jejunum Ileum and Colon and Cecum. Images were taken with a 40x objective
**Supplemental Figure 4: Biodistribution of HA‐647 in Intestines:** Representative ex vivo fluorescence image obtained with IVIS of GI organs (A) 0.08 h and (B) 0.5 h after I.V. administration of HA conjugated with alexafluor 647. Organs: (1) Stomach; (2) Duodenum; (3) Jejunum; (4) Ileum; (5) Cecum; and (6) Colon. A scale of the radiance efficiency is presented to the right of excised mouse organ image.
**Supplemental Figure 5: Biodistribution of HA‐Dox‐647 in Intestine:** Representative ex vivo fluorescence image obtained with IVIS of GI organs (A) 0.08 h and (B) 0.5 h after I.V. administration of HA‐Dox conjugated with Alexafluor 647. Organs (1) Stomach; (2) Duodenum; (3) Jejunum; (4) Ileum; (5) Cecum; (6) Colon. A scale of the radiance efficiency is presented to the right of excised mouse organ image. Representative flow cytometric analysis of Dox presence in (C) Duodenum and Jejunum (D) Ileum and Colon (E) Cecum homogenates 0.08 h and 0.5 h after I.V. administration
**Supplemental Figure 6:** Inflammation analysis of Murine Intestinal Tissues Induced with Chemical Induced Colon Cancer: Immunohistochemistry of Intestinal Tissues treated with azoxymethane and dextran sulfate treated with saline, Dox, and HA‐Dox, determined by (A) Cox‐2 and (B) iNOS staining. Representative images were taken at 400x. Red scale bar: 50 um
**Supplemental Figure 7:** Apoptotic analysis of Murine Intestinal Tissues Induced with Chemical Induced Colon Cancer: Immunohistochemistry of Intestinal Tissues treated with azoxymethane and dextran sulfate treated with saline, Dox, and HA‐Dox, determined by (A) Caspase‐3 and (B) Bax staining. Representative images were taken at 400x. Red scale bar: 50 um
