# Supplementary material for: Hyaluronic acid–doxorubicin nanoparticles for targeted treatment of colorectal cancer
Source: Bioeng Transl Med. 2020 May 28;6(1):e10166. doi: 10.1002/btm2.10166 (PMC7823125; doi:10.1002/btm2.10166)
Supplement: Supplementary file 1 — Supplemental Figure 1 Accumulation of HA‐Dox in Perfused Intestines:. The amount of HA‐Dox represented as percentage of injected dose (%ID) for free Dox and HA‐Dox following I.V. injection in mice at 24 h after perfusion compared to no perfusion remaining in different parts of murine intestine. Each data point represent means ± SEM (n = 3).*, p < 0.05; non paired, two‐tailed t‐test. Supplemental Figure 2: Accumulation of HA‐Dox in Intestines: Representative flow cytometry histograms portraying the presence of HA‐Dox following I.V. injection in mice at 24 h in (A) Duodenum and Jejunum (B) Ileum and Colon and (C) Cecum homogenates. Dox spiked in was used as a positive control. Supplemental Figure 3: Accumulation of HA‐Dox in Intestines: Representative confocal images portraying the presence of HA‐Dox represented by dots following I.V. injection in mice at (A) 0.08 h and (B) 6 h in Duodenum and Jejunum Ileum and Colon and Cecum. Images were taken with a 40x objective Supplemental Figure 4: Biodistribution of HA‐647 in Intestines: Representative ex vivo fluorescence image obtained with IVIS of GI organs (A) 0.08 h and (B) 0.5 h after I.V. administration of HA conjugated with alexafluor 647. Organs: (1) Stomach; (2) Duodenum; (3) Jejunum; (4) Ileum; (5) Cecum; and (6) Colon. A scale of the radiance efficiency is presented to the right of excised mouse organ image. Supplemental Figure 5: Biodistribution of HA‐Dox‐647 in Intestine: Representative ex vivo fluorescence image obtained with IVIS of GI organs (A) 0.08 h and (B) 0.5 h after I.V. administration of HA‐Dox conjugated with Alexafluor 647. Organs (1) Stomach; (2) Duodenum; (3) Jejunum; (4) Ileum; (5) Cecum; (6) Colon. A scale of the radiance efficiency is presented to the right of excised mouse organ image. Representative flow cytometric analysis of Dox presence in (C) Duodenum and Jejunum (D) Ileum and Colon (E) Cecum homogenates 0.08 h and 0.5 h after I.V. administration Supplemental Figure 6: Inflammation analysis [file BTM2-6-e10166-s001.docx]

**Supplemental Figures**


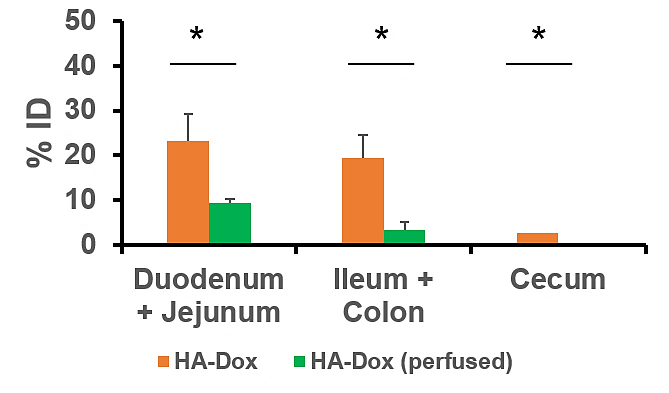


**Supplemental Figure 1: Accumulation of HA-Dox in Perfused Intestines:**. The amount of HA-Dox represented as percentage of injected dose (%ID) for free Dox and HA-Dox following I.V. injection in mice at 24 h after perfusion compared to no perfusion remaining in different parts of murine intestine. Each data point represent means ± SEM (n=3).*, P< 0.05; non paired, two-tailed t-test.


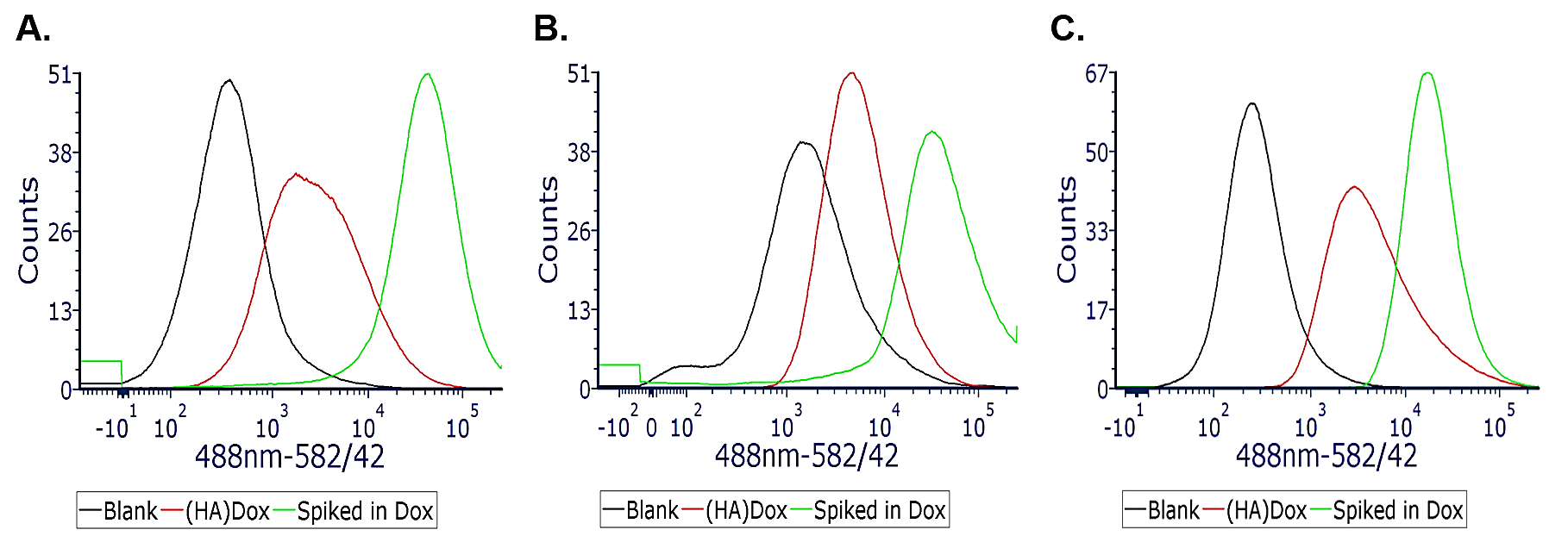


**Supplemental Figure 2: Accumulation of HA-Dox in Intestines:** Representative flow cytometry histograms portraying the presence of HA-Dox following I.V. injection in mice at 24 h in (A) Duodenum and Jejunum (B) Ileum and Colon and (C) Cecum homogenates. Dox spiked in was used as a positive control.


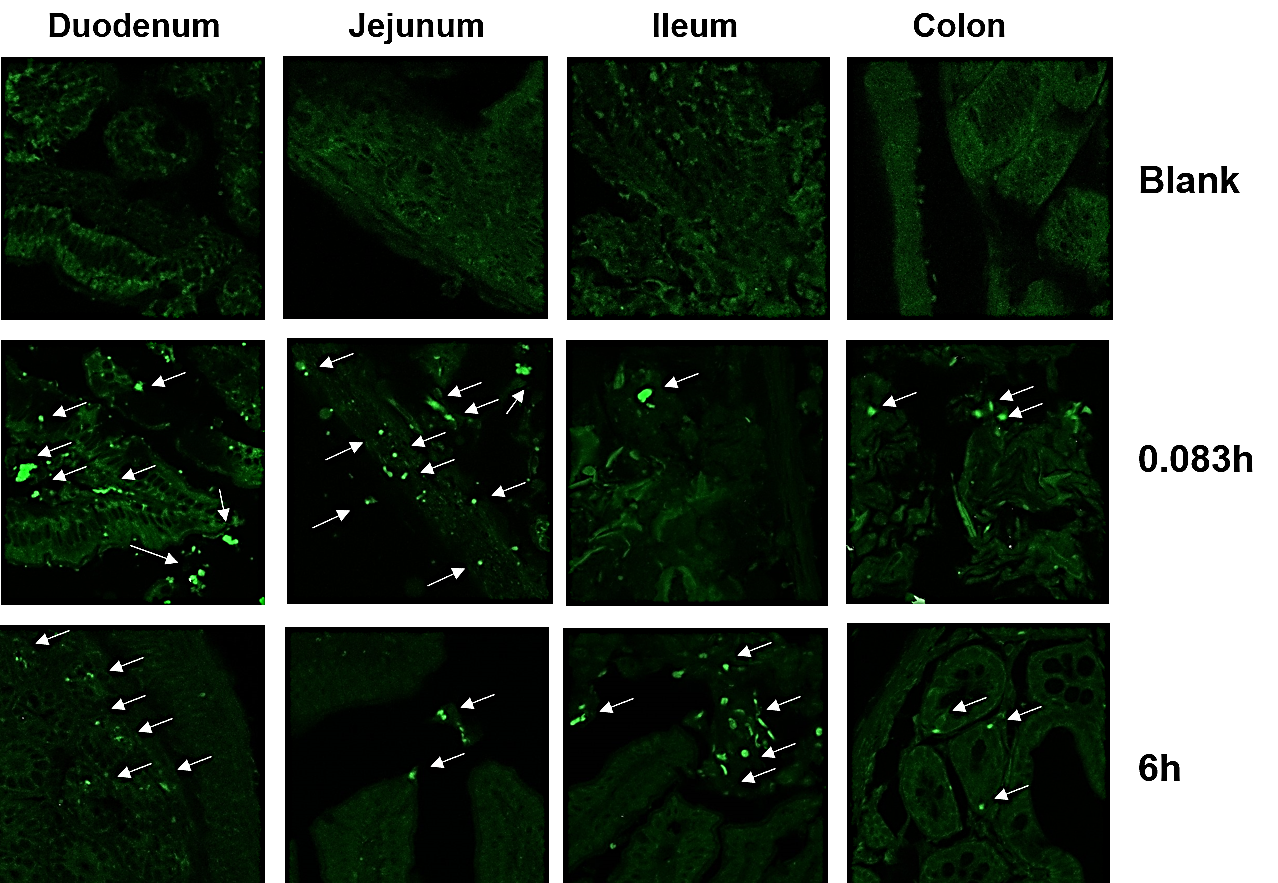


**Supplemental Figure 3: Accumulation of HA-Dox in Intestines:** Representative confocal images portraying the presence of HA-Dox represented by dots following I.V. injection in mice at (A) 0.08 h and (B) 6 h in Duodenum and Jejunum Ileum and Colon and Cecum. Images were taken with a 40x objective


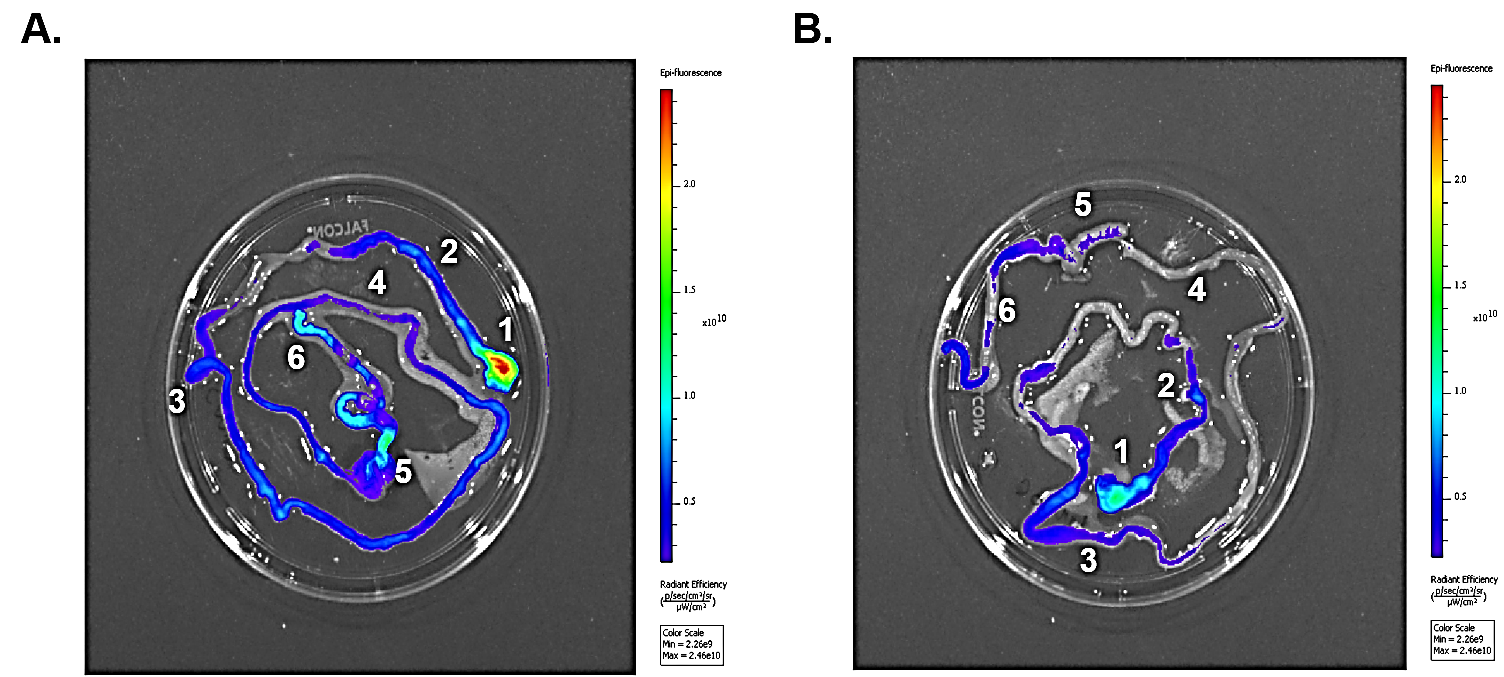


**Supplemental Figure 4: Biodistribution of HA-647 in Intestines:** Representative *ex vivo* fluorescence image obtained with IVIS of GI organs (A) 0.08 h and (B) 0.5 h after I.V. administration of HA conjugated with alexafluor 647. Organs: (1) Stomach; (2) Duodenum; (3) Jejunum; (4) Ileum; (5) Cecum; and (6) Colon. A scale of the radiance efficiency is presented to the right of excised mouse organ image.


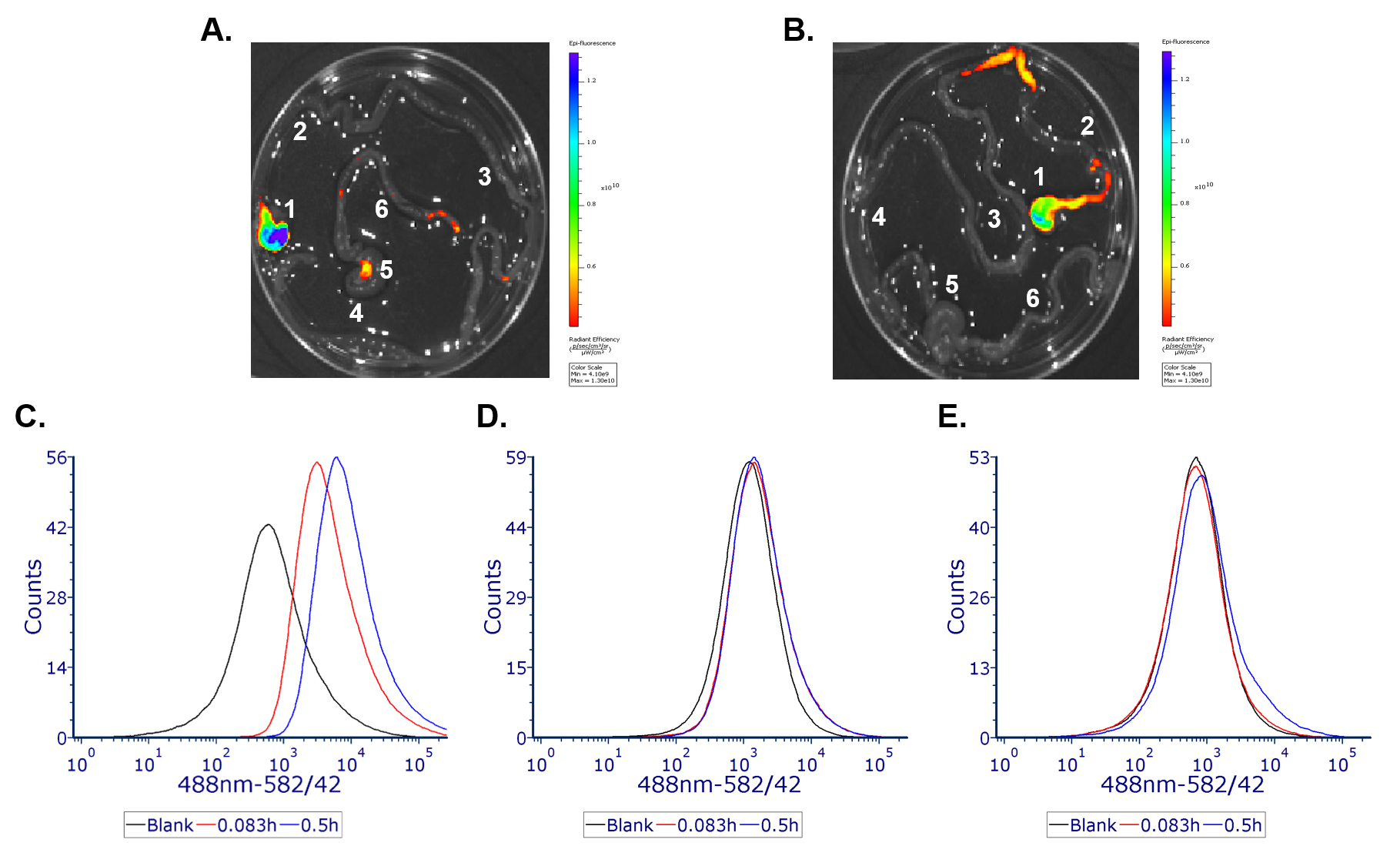


**Supplemental Figure 5: Biodistribution of HA-Dox-647 in Intestine:** Representative *ex vivo* fluorescence image obtained with IVIS of GI organs (A) 0.08 h and (B) 0.5 h after I.V. administration of HA-Dox conjugated with Alexafluor 647. Organs (1) Stomach; (2) Duodenum; (3) Jejunum; (4) Ileum; (5) Cecum; (6) Colon. A scale of the radiance efficiency is presented to the right of excised mouse organ image. Representative flow cytometric analysis of Dox presence in (C) Duodenum and Jejunum (D) Ileum and Colon (E) Cecum homogenates 0.08 h and 0.5 h after I.V. administration


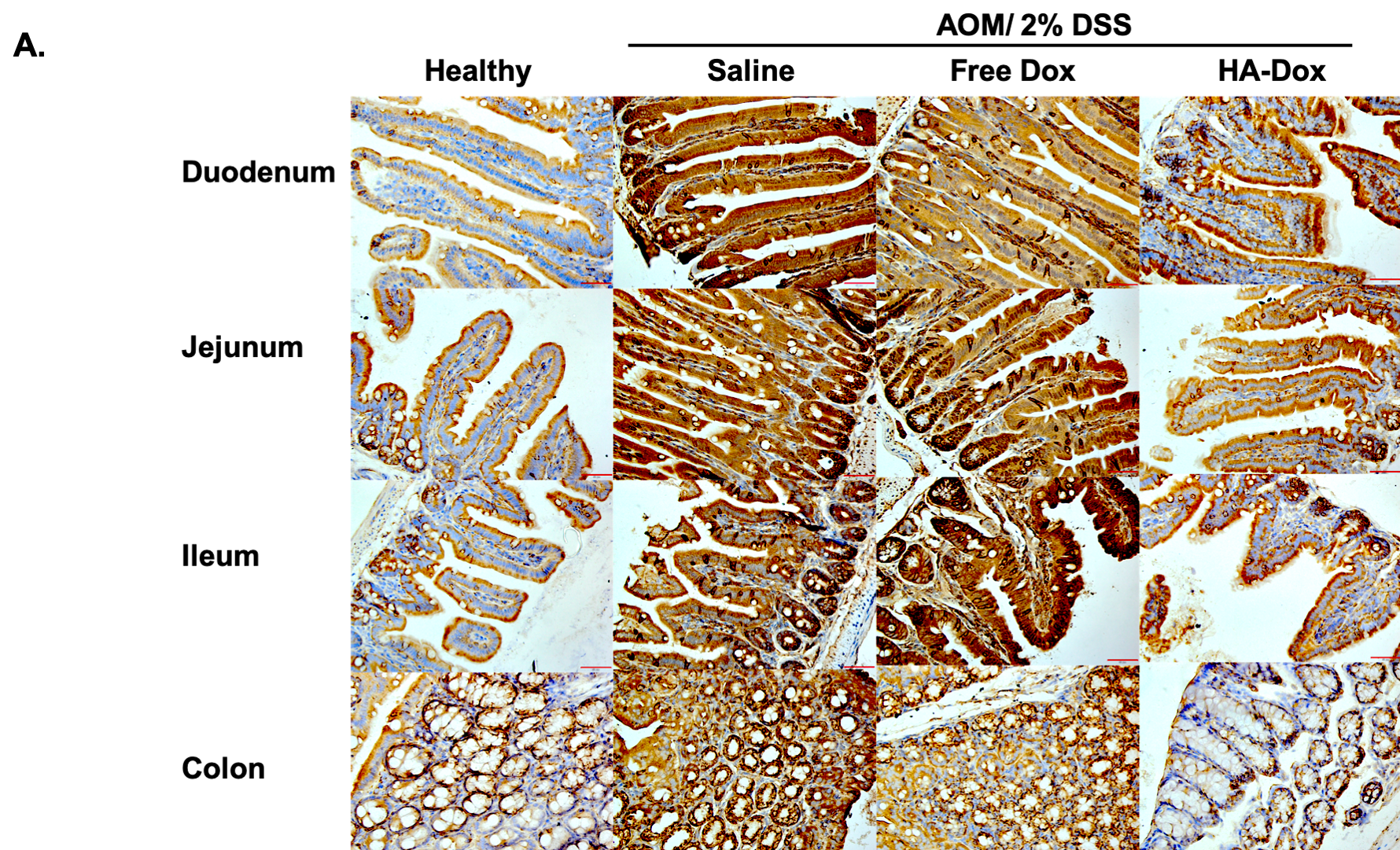


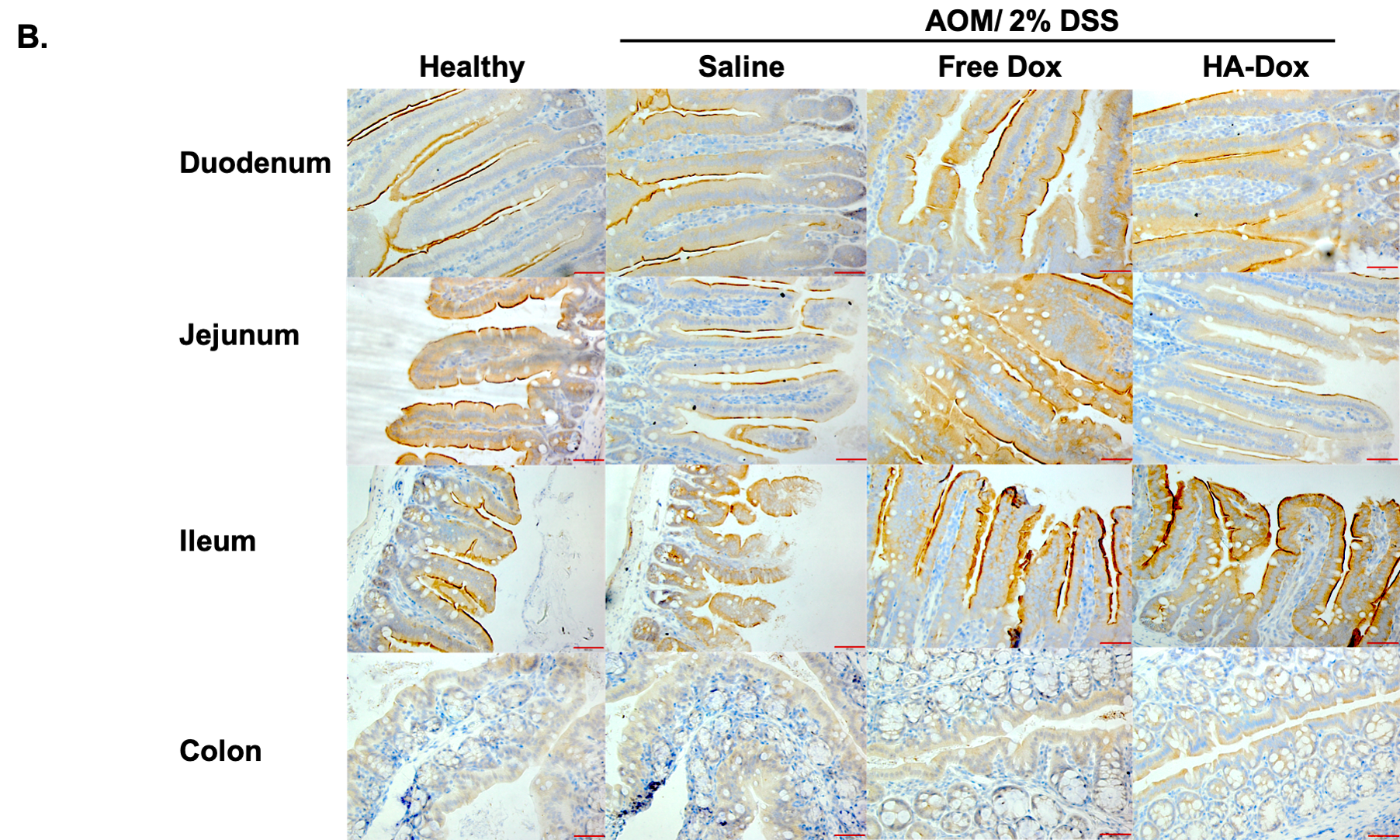


**Supplemental Figure 6:** Inflammation analysis of Murine Intestinal Tissues Induced with Chemical Induced Colon Cancer: Immunohistochemistry of Intestinal Tissues treated with azoxymethane and dextran sulfate treated with saline, Dox, and HA-Dox, determined by (A) Cox-2 and (B) iNOS staining. Representative images were taken at 400x. Red scale bar: 50 um


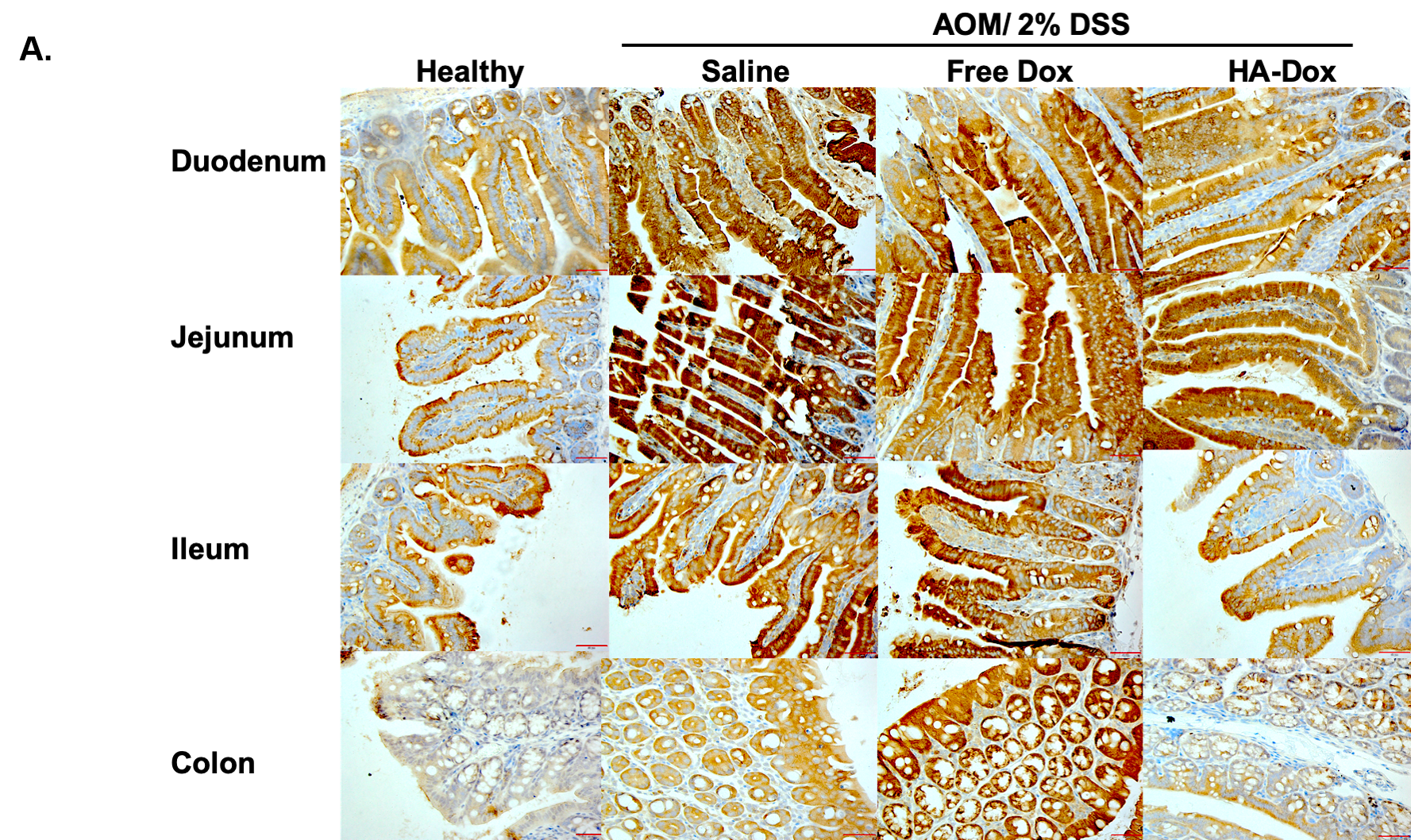


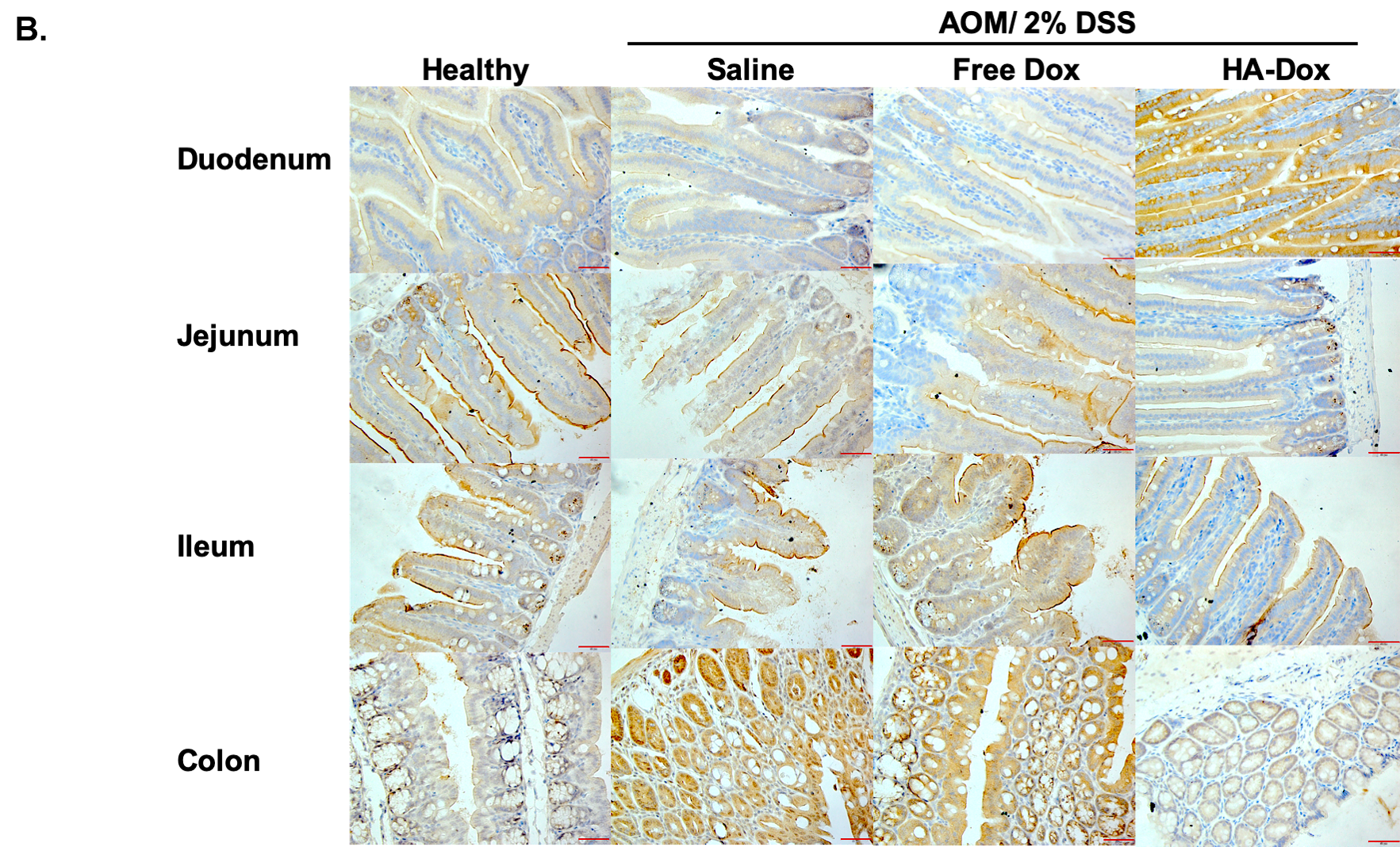


**Supplemental Figure 7:** Apoptotic analysis of Murine Intestinal Tissues Induced with Chemical Induced Colon Cancer: Immunohistochemistry of Intestinal Tissues treated with azoxymethane and dextran sulfate treated with saline, Dox, and HA-Dox, determined by (A) Caspase-3 and (B) Bax staining. Representative images were taken at 400x. Red scale bar: 50 um
